# Introducing the Truth Effect Database (TED): An open trial-level resource promoting FAIR data in truth effect research

**DOI:** 10.3758/s13428-026-03007-y

**Published:** 2026-04-16

**Authors:** Sven Lesche, Annika Stump

**Affiliations:** 1 Ruprecht-Karls-University Heidelberg, Hauptstraße 47-51, 69117 Heidelberg, Germany; 2 Albert-Ludwigs-University Freiburg, Freiburg Im Breisgau, Germany

**Keywords:** Illusory truth effect, Open science, Open data, Database, SQL, Reproducibility

## Abstract

The FAIR (Findable, Accessible, Interoperable, and Reusable) data principles form the foundation of the open data movement. However, while many current practices ensure data are findable and accessible, true interoperability and reusability remain limited. This paper introduces the Truth Effect Database (TED), a large-scale, trial-level, open database harmonizing data from illusory truth effect studies designed to enhance interoperability and reusability. TED currently integrates data from 59 studies in 29 publications, spanning 12,249 participants and 808,231 trials, accounting for a wide range of dispositional and contextual variables. To promote usability, TED focuses on user-friendly data submission using a custom entry website and data extraction using the R package *acdcquery.* These tools guide researchers through both data entry and retrieval, eliminating the need for direct interaction with the database’s internal structure. We illustrated the utility of TED through Bayesian multilevel analyses, highlighting substantial variance in the illusory truth effect at the subject level, moderated by the delay between exposure and judgment phases in truth effect paradigms. Beyond this first demonstration, TED provides the foundation for a wide range of future research. These include (living) meta-analyses, simulation-based power analyses, rigorous replication and reanalysis of existing studies, and the validation and development of formal cognitive models. As an open and extensible infrastructure, TED serves as a blueprint for sustainable, community-driven database development in psychological science.

The open science movement has emerged in response to growing concerns about the replicability, transparency, and efficiency of scientific research (Ioannidis, 2005; Munafò et al., 2017; Nosek et al., 2015). At its core, open science promotes practices that make research processes and outputs accessible, verifiable, and reusable. Practices such as preregistration, data sharing, and open materials aim to increase the trustworthiness of findings and to foster a more cumulative, collaborative scientific enterprise. These practices, coupled with platforms like the Open Science Framework (OSF; Foster & Deardorff, 2017), have played a pivotal role in advancing openness through tools that support study registration, version control, and public sharing of data and materials. Transparency, once rare, is increasingly becoming the norm (Bauer, 2022).

However, while transparency has improved markedly, efficiency and reusability have lagged. Research materials and datasets are often siloed within individual project pages, tailored to host single studies. As a result, even when data are technically open, they are rarely standardized in ways that facilitate reuse, integration, or cumulative analysis. Current open data practices focus primarily on the first two aspects of the FAIR (Findable, Accessible, Interoperable, and Reusable) principles of data sharing (Wilkinson et al., 2016) while neglecting the latter. For example, the OSF is well suited to documenting and finding individual projects, but its structure does not support the aggregation or harmonization of data across multiple related studies. In addition to structural limitations, researchers’ practices like inconsistent file formats, project-specific codebooks, and divergent analytic pipelines continue to hinder interoperability.

This inconsistency limits not only replicability, but also reusability. Should a researcher be interested in estimating an effect size, developing a new computational model, or simply reanalyzing existing data instead of collecting an entirely new sample to investigate a further research question, they have to comb through OSF, pray for existing codebooks, and hope for decipherable raw data. This is both tedious and often unsuccessful (Crüwell et al., 2023; Hardwicke et al., 2021). However, reusability greatly improves scientific efficiency. It saves resources otherwise wasted by planning and collecting an entirely new set of data and helps efficiently allocate public resources as well as participants’ and researchers’ time.

We believe that structure is essential to make research results not only transparent, but also truly reusable. That is, reusability requires standardized organization: consistent variable naming, common data schemas, uniform documentation practices, and clear, accessible codebooks. In neuroscience, this principle has been successfully realized through the Brain Imaging Data Structure (BIDS) (Gorgolewski et al., 2016), which provides a framework for organizing and annotating neuroimaging data. BIDS has enabled not just more efficient sharing, but also reproducible pipelines, automated analyses, and collaborative efforts at scale (Poldrack et al., 2024).

Structured approaches like BIDS are now gaining increasing attention in cognitive psychology. The Attentional Control Data Collection (ACDC) introduced a trial-level database for data from attentional control experiments, providing standardized variable names, metadata, and analysis-ready formats that streamline cross-study comparisons (Haaf et al., 2025). Similarly, the FEARBASE project is building a large-scale, open-access repository for fear conditioning studies, adopting a shared structure to ensure comparability and long-term usability (Lonsdorf & Ehlers, 2025). Crucially, there is increasing institutional support for the development of infrastructure enabling data reuse. For example, the German research foundation DFG issued an open funding call for projects developing infrastructure for scientific data management (DFG; Förderprogramm Informationsinfrastrukturen für Forschungsdaten, 2025). These projects spearhead a changing culture of data reusability but so far represent the exception rather than the norm.

We believe that there is great potential in the development of structured databases integrating trial-level data in the field of cognitive psychology. Large and structured collections of raw data have several key applications, extending the general principle of data reusability. They enable living meta-analyses that can update automatically as new data are added, allow researchers to find relevant raw data based on task characteristics or participant variables, facilitate the straightforward replication of published findings, and enable power analyses using pooled datasets from comparable studies. Beyond these direct applications, structured datasets also support the development of new methods and models. Trial-level repositories allow for benchmarking existing analytic tools, training machine learning models, and exploring novel statistical approaches.

To illustrate the potential of such structured and reusable databases, we focus on a particularly relevant and empirically rich phenomenon in cognitive and social psychology: the illusory truth effect (ITE). The ITE has been robustly demonstrated across numerous studies, yet substantial heterogeneity in effect sizes, experimental designs, and individual-level moderators complicates efforts to draw generalizable conclusions. These complexities make it an ideal case for a centralized, harmonized repository of trial-level data. In the following section, we provide a brief overview of the ITE and explain how its empirical challenges underscore the need for exactly the kind of infrastructure we propose.

## The illusory truth effect

The ITE exemplifies a psychological mechanism with both theoretical significance and pressing real-world relevance. In today’s data-saturated and fast-paced information environment, individuals are routinely exposed to repeated content of uncertain credibility—from benign repetition to coordinated misinformation. As such, understanding how repetition influences perceived truth is central to addressing challenges in belief formation, misinformation spread, and public trust. Originally demonstrated by Hasher et al. (1977), the ITE refers to the tendency to perceive repeated information as more credible than novel ones, regardless of their actual accuracy. Since then, the effect has been robustly replicated in over 80 studies.

Previous research indicates that the illusory truth effect is primarily driven by increased processing fluency as a result of repetition. That is, when evaluating the truthfulness of a statement, individuals often draw on their subjective sense of ease during processing as a heuristic cue (e.g., Dechêne et al., 2010). Crucially, the relative fluency appears to be decisive: Dechêne et al. (2009) showed that the effect only reliably occurs when fluent and disfluent stimuli are presented together. In experimental paradigms investigating the ITE, fluency is typically manipulated by contrasting new statements with repeated ones which have been shown during a prior exposure phase. Although ITE has been observed across various domains and populations, prior research indicates that the illusory truth effect significantly varies among individuals (e.g., Nadarevic, 2010; Schnuerch et al., 2021).

Research examining dispositional factors that may account for individual differences in susceptibility to the illusory truth effect has yielded mixed and often inconclusive findings. For example, Arkes et al. (1991) and Boehm (1994) investigated need for cognition (NFC) as a potential trait moderator of the effect but found no supporting evidence. Similarly, Newman et al. (2020) explored whether NFC moderates the illusory truth effect and found that this relationship was contingent upon the nature of the experimental instructions. Specifically, the moderating influence of NFC emerged only when participants were not informed that the exposure phase contained both true and false statements; when such information was provided, the effect was no longer evident. In addition to NFC, other dispositional factors have been investigated with similarly inconsistent results. For instance, Kim (2002) examined whether skepticism moderates the effect and reported ambiguous findings, while DiFonzo et al. (2016) found marginal evidence for such moderation. Sundar et al. (2015), focusing on repeated health-related messages, observed that individuals with a high need for affect (i.e., the tendency to approach or avoid affect-inducing situations) were more susceptible to the ITE. More recently, De Keersmaecker et al. (2020) tested three further individual difference variables—need for cognitive closure (NCC), thinking style preference (intuitive vs. deliberative), and cognitive ability—but found no evidence that any of these measures moderated the truth effect. In two experiments, Stump et al. (2022) found evidence suggesting moderating effects for NCC on the illusory truth effect after a relatively short but not after a longer retention interval (10 min vs. 1 week). Crucially, De Keersmaecker et al. (2020) assessed NCC exclusively in one study, which employed a single, relatively long retention interval of 5–7 days. The pattern of findings suggests that the temporal distance between exposure and judgment phase may be a key factor when assessing the role of individual differences in the truth effect. Further support for this interpretation comes from recent work by Schnuerch et al. (2021), who conducted a comprehensive Bayesian reanalysis of multiple datasets examining individual differences in the illusory truth effect. Their results revealed robust evidence for such differences in five datasets, whereas at least two datasets provided evidence against them. Crucially, in the latter two studies, the retention interval spanned 1 week, while in the other datasets, the judgment phase occurred within the same experimental session as the exposure phase.

Taken together, these findings offer preliminary insights into how individual differences may shape susceptibility to the truth effect. However, they also highlight the complexity of the phenomenon and suggest that an integrative approach—considering both dispositional and contextual variables—is essential for a more comprehensive understanding of the mechanisms underlying the ITE.

## The present study

In the spirit of providing FAIR data to aid research on ITE, we introduce a centralized, trial-level database, drawing on resources and lessons learned from developing ACDC (Haaf et al., 2025). The Truth Effect Database (TED) is designed not only with structured organization in mind, but also with an emphasis on ease of use. Particular attention has been given to lowering the barrier for data submission through an intuitive entry mask, as well as to enabling simple data extraction via an R package. Our aim is to create a living, extensible resource that supports both contributors and users: researchers can add new studies with minimal friction, and others can search, filter, and analyze trial-level data without having to clean or realign disparate datasets. By combining structure with usability, this resource is intended to foster cumulative research on the illusory truth effect and to serve as a model for reusable psychological data infrastructures more broadly. The accompanying website (https://slesche.github.io/ted-site/) aims to serve as both a guide for using TED and a living example of analyses made possible through TED. In addition to a living version of the analysis provided in this paper, we provide a small, living meta-analysis—in fact, the most recent meta-analysis published in the field of the illusory truth effect dates back more than 15 years (see Dechêne et al., 2010).

## Method

### The Truth Effect Database (TED)

Resources to build the database, restructure raw data, build the website, and integrate the submitted data can be found on GitHub (https://github.com/SLesche/truth-effect-database). We also provide a landing page with detailed information on the project and a user guide for interacting with the database (https://slesche.github.io/ted-site/).

The database is implemented using Structured Query Language (SQL), specifically *SQLite* (Hipp, 2020). A key advantage of SQLite is its serverless architecture—data are stored in a single, shareable file, allowing researchers to download, interact with, and modify the database locally without the need for a dedicated database server.

As a relational database system, SQLite supports the use of multiple interconnected tables. This structure enables efficient organization of complex data relationships. For instance, each publication may include several studies, which themselves involve multiple experimental conditions. By organizing data across discrete, normalized tables rather than in a single flat file, TED minimizes redundancy and enhances both storage efficiency and query performance.

**Fig. 1:**
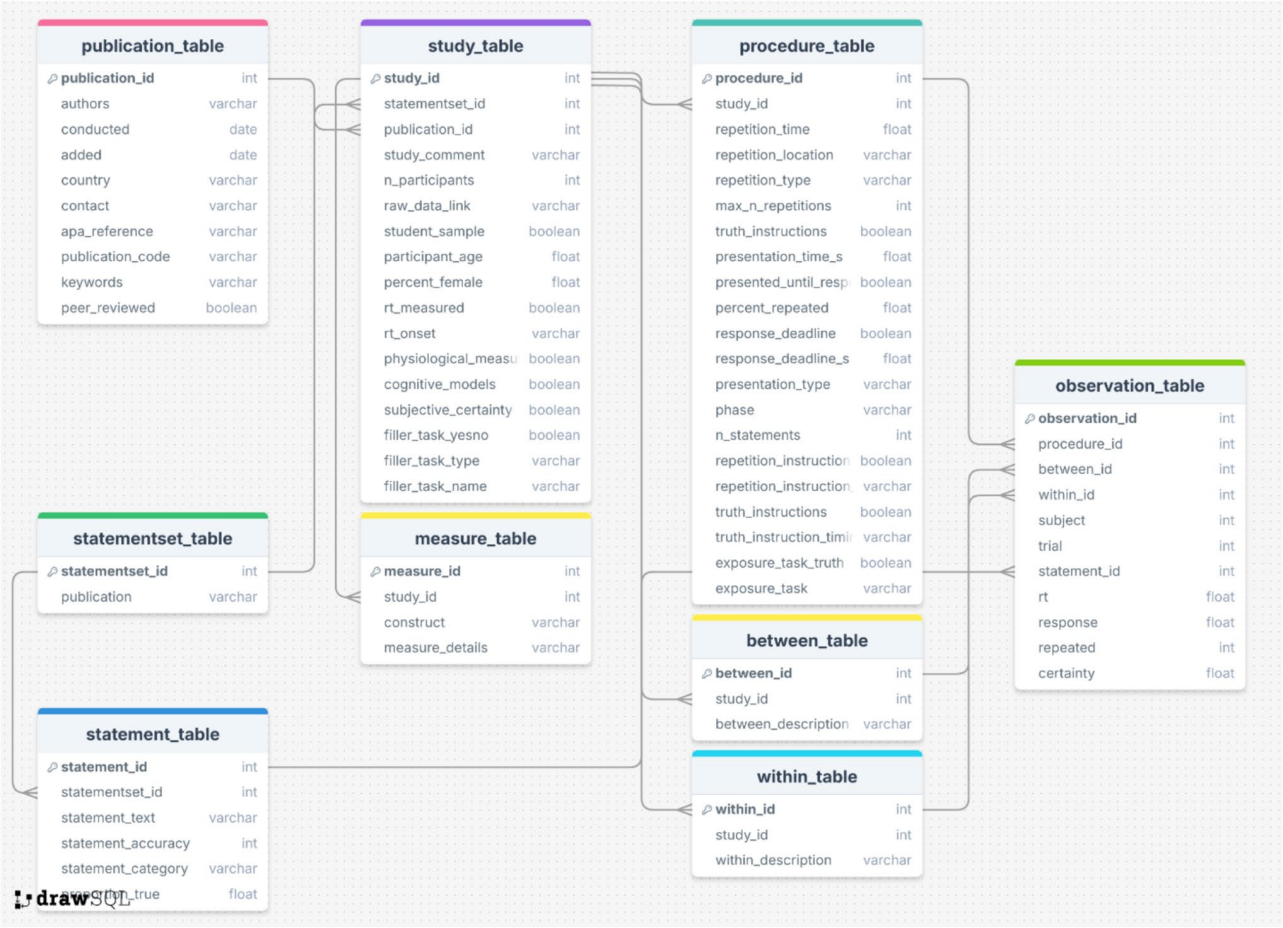
Overview of TED structure. *Note.* Structure of TED. The overview displays all tables with their primary keys indicated in bold, as well as the references between tables through lines connecting variables across data. Furthermore, the type of each variable is shown (integer – int, character – varchar, float – float, or boolean – Boolean)

The design of our database is illustrated in Fig. 1. Broadly, we make use of a table for each part of data related to truth effect experiments. Metadata concerning the publications themselves as the highest order are stored in a publication_table, and raw data at the lowest level in the observation_table. Between these two levels, additional tables capture detailed information about studies, procedures, materials, and collected measures (see Table 1).

**Table 1:** Overview of tables in TED

Table	Description
*publication_table*	Contains metadata about the publication (e.g., authors, year, journal)
*study_table*	Includes study-level information such as participant characteristics, use of filler tasks, and the location of raw data in online repositories
*procedure_table*	Describes the experimental procedure, including commonly manipulated variables in truth effect research (e.g., type of repetition, delay between exposure and judgment phase, warnings about the truth effect)
*between_table*	Lists between-subject manipulations not already encoded in the *procedure_table*
*within_table*	Lists within-subject manipulations not already encoded in the *procedure_table*
*statementset_table*	Provides information about the publication or source from which a set of statements was retrieved
*measure_table*	Includes information about additional measures collected during the experiment
*observation_table*	Stores the harmonized raw trial-level data, representing the lowest level of the database structure

This relational approach provides a clear and modular organization of data sources, linking tables through unique identifier variables. We argue that while the use of a relational database adds some complexity, it also introduces an intuitive naming system and structure for variables of interest. Importantly, our goal is that variable names and their table location are the only knowledge that users need to have in order to interact with the database. To this end, we have developed tools that require little to no understanding of the database structure or SQL in order to submit and extract data from the database.

#### Data submission

**Fig. 2:**
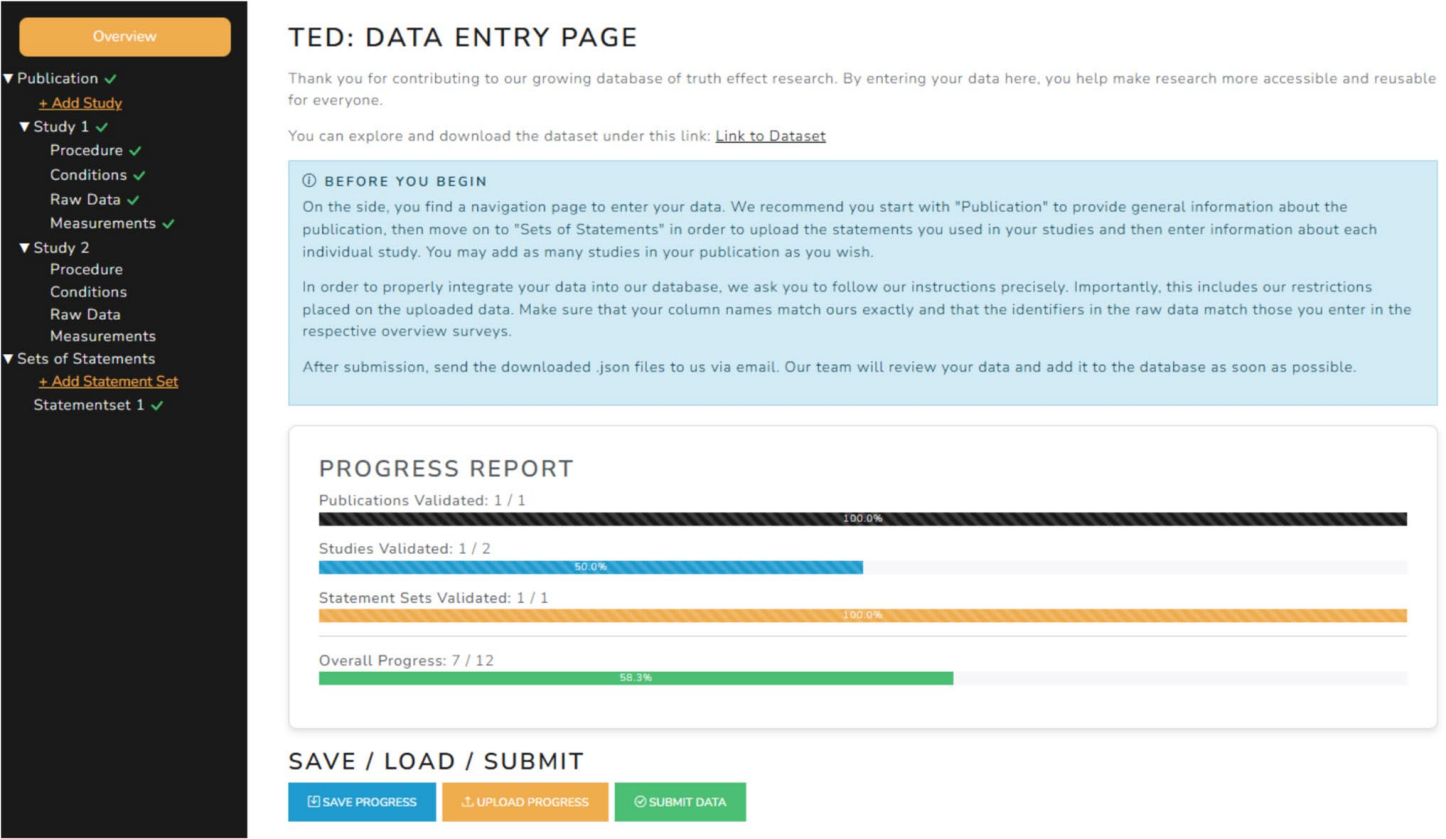
Screenshot of the TED data entry webpage. *Note.* Overview page of the TED data submission website. Submission information is entered in sequential stages via the left-hand sidebar and automatically validated to ensure compliance with TED formatting requirements. After completing all required sections, researchers can generate a finalized submission file by clicking “Submit Data” and send this file to the TED maintainers via email for review and integration

To ease the process of submitting data to the database, we built a website (https://slesche.github.io/truth-effect-database/) that guides users through the submission process and checks submitted information for inconsistencies or errors (see Fig. 2). This website is designed to both minimize the effort of submitting new data to the database and proactively identify potential errors in the submission process.

Contributors are prompted to provide information across all levels of the database, including publication metadata, study characteristics, experimental conditions, statements, and raw trial data. Importantly, this also requires formatting the raw data according to the documented TED variable names. By placing the responsibility for mapping and labeling variables on the researchers most familiar with the dataset, ambiguities about variable meaning are resolved at the source, and the data are directly translated into a standardized format that is transparent and usable for the broader community.

The system cross-checks entered metadata against the uploaded raw data (e.g., verifying the number and coding of conditions or statements) and provides immediate feedback if inconsistencies arise. These automated validation steps ensure that submissions are internally coherent and can be smoothly integrated into the harmonized database structure, while maintaining a low barrier to contribution.

Once all required information has been entered and validated, contributors finalize their submission by clicking the “Submit Data” button. This action generates and downloads a finalized JSON file containing the complete submission package. Contributors then send this file to the TED maintainers for review and final approval, after which the data are integrated into the database.

Data can be submitted at any stage of the research process, either immediately after data collection, upon release of a preprint, or following formal publication. If the status of the associated manuscript changes, contributors can easily update this information by contacting the TED maintainers and informing them of the new status.

#### Data extraction

We extended the R package *acdcquery* (Lesche, 2025) to simplify the process of extracting data. This package provides functions to facilitate connection to the database and extraction of data. Users can download the latest version of the database using download_ted(), connect to the database using connect_to_db(), define filter arguments using add_argument(), and request specific variables from any table in the database using query_db().

For example, users may request all available trial-level data from studies with greater than 200 participants from the judgment phase using the code below:
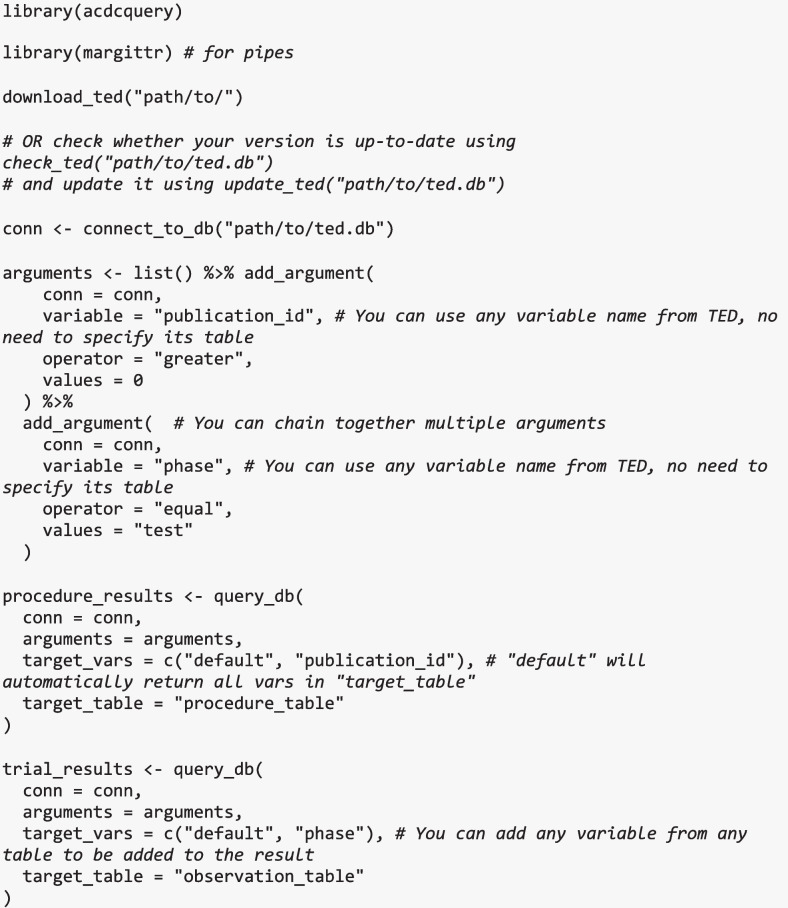


The R package will automatically construct the necessary SQL query and return the filtered data with the selected columns to the user. A detailed user guide on using the R package to interact with our database can be found on the database’s GitHub (https://github.com/SLesche/truth-effect-database).

#### Data selection

We included only data from studies specifically focused on the repetition-based illusory truth effect. To qualify for inclusion, studies had to involve participants making truth judgments about visual or auditory stimuli, with a subset of those stimuli having been presented previously to allow for repetition effects. Our data selection process was based in part on the work of Henderson et al. (2022), who identified 17 publications with accessible raw data. Additionally, we contacted colleagues directly to request openly available datasets, further expanding the scope of included studies and conducted a literature search to find open data from the past years.

#### Data analysis

To illustrate the analytical potential of TED, we conducted a Bayesian multilevel model predicting truth judgments, incorporating both fixed and random effects of repetition at the statement, subject, and procedure (i.e., experimental) levels. The scale and structure of TED enable us to estimate the variance in the repetition effect simultaneously across all three levels. This approach allows us to determine whether the size of the truth effect varies more substantially across individuals, across experimental procedures, or across the specific statements used.

## Results

**Fig. 3:**
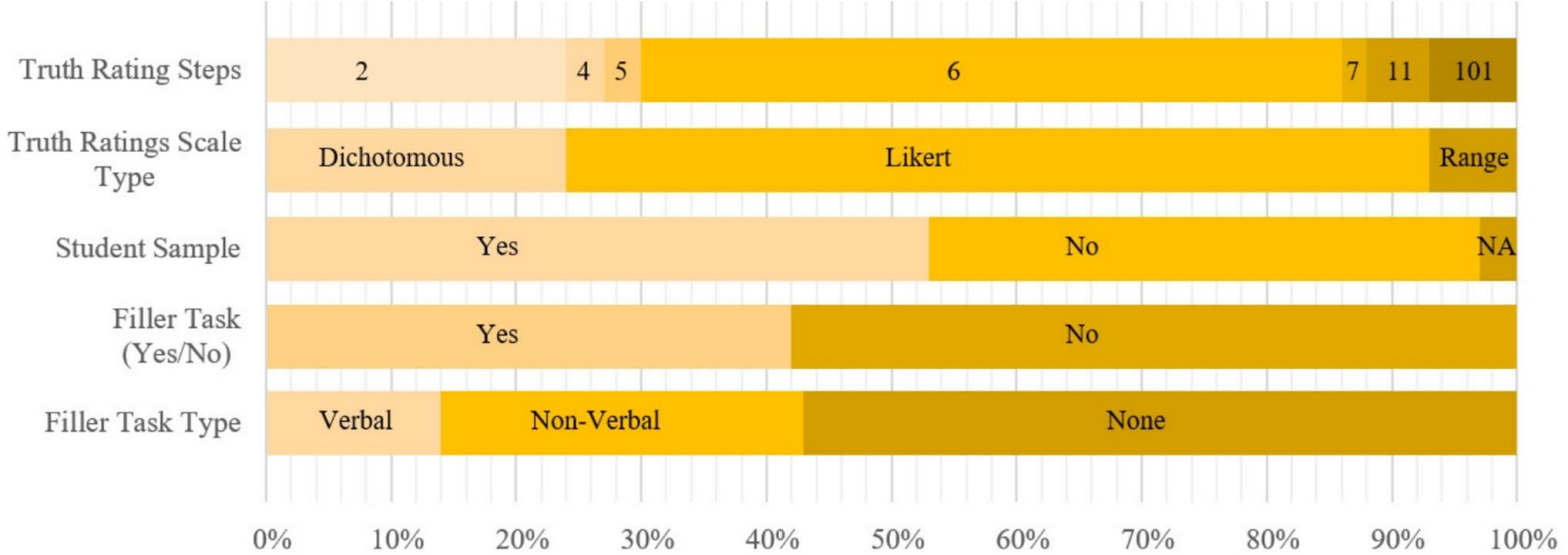
Overview of study-related variables in TED. *Note.* Overview of *study_table* variables highlighting variability in key ITE design features across studies included in TED to date. “Truth Ratings Steps” indicates the number of response options used for truth judgments (ranging from binary judgments to 0–100 sliders). “Truth Rating Scale Type” specifies whether the scale was dichotomous, Likert-type, or continuous. “Student Sample” indicates whether the sample consisted of university students. “Filler Task (Yes/No)” and “Filler Task Type” indicate whether a filler task was administered between the exposure and judgment phases, and whether this task was verbal or nonverbal. Variable labels in the figure were slightly simplified for readability and may differ from the exact variable names in the database (e.g., “Truth Ratings Steps” vs. *truth_ratings_steps*)

All analysis was conducted using R [Version 4.4.1; R Core Team (2022)].^1^ In the current version of the manuscript, we included 59 studies from 29 publications, spanning 12,249 participants contributing 808,231 trials. A complete list of the included publications can be found in Table 2. Sample composition ranged from 29 to 949 participants. On average, studies included 211.24 participants (${\mu }_{age}=$ 32.92,${\sigma }_{age}=$ 7.33). An overview of the rating scale usage for truth judgments as well as the use of a filler task over all included studies can be found in Fig. 3.

**Table 2:** Publications included in TED

publication_id	authors	peer_reviewed	study_id	n_participants	n_trials
1	Bena et al. (2019)	No	1	186	10,416
2	Bena et al. (2020)	No	2	138	4,968
3	Brashier et al. (2020)	Yes	3	103	12,480
3		Yes	4	99	19,800
3		Yes	5	68	13,600
3		Yes	6	89	18,600
4	Bena et al. (2022)	Yes	7	380	15,200
5	De Keersmaecker et al. (2024)	Yes	8	283	5,320
5		Yes	9	271	5,200
5		Yes	10	200	11,520
5		Yes	11	299	11,960
5		Yes	12	291	11,680
6	De Keersmaecker et al. (2025)	No	13	113	22,600
6		No	14	430	86,000
7	Ecker et al. (2020)	Yes	15	371	4,476
7		Yes	16	939	11,376
7		Yes	17	408	4,932
8	Fazio et al. (2019)	Yes	18	503	40,240
9	Hatzidaki et al. (2025)	Yes	19	82	9,840
9		Yes	20	68	8,160
10	Henderson et al. (2021)	Yes	21	507	68,800
11	Jalbert et al. (2020)	Yes	22	220	15,840
11		Yes	23	282	20,376
11		Yes	24	405	29,088
12	Lacassagne et al. (2022)	Yes	25	240	3,712
13	Lorenzoni et al. (2024)	Yes	26	60	4,960
14	Murray et al. (2020)	No	27	526	48,825
15	Nadarevic (2007)	No	28	54	9,504
16	Nadarevic and Rinnewitz (2011)	No	29	139	2,780
17	Nadarevic et al. (2012)	No	30	267	5,340
18	Nadarevic and Erdfelder (2014)	Yes	31	85	22,968
18		Yes	32	66	6,072
19	Nadarevic and Aßfalg (2017)	Yes	33	65	11,440
19		Yes	34	202	14,000
20	Nadarevic et al. (2018)	Yes	35	73	4,088
20		Yes	36	79	8,848
21	Nadarevic et al. (2020)	Yes	37	91	5,460
21		Yes	38	64	3,840
21		Yes	39	80	4,320
22	Nadarevic et al. (2021)	Yes	40	70	11,200
22		Yes	41	149	26,820
22		Yes	42	98	4,800
23	Nadarevic and Erdfelder (2025)	Yes	43	64	5,376
23		Yes	44	64	5,376
23		Yes	45	65	5,200
24	Newman et al. (2020)	Yes	46	89	6,624
25	Pennycook et al. (2018)	Yes	47	409	11,452
25		Yes	48	949	22,776
25		Yes	49	940	28,624
26	Stump et al. (2022)	Yes	50	97	11,640
26		Yes	51	75	9,000
27	Stump et al. (2024)	Yes	52	81	8,850
28	Unkelbach and Greifeneder (2018)	Yes	53	29	3,240
28		Yes	54	41	4,920
28		Yes	55	42	5,040
28		Yes	56	37	3,000
29	Vogel et al. (2020)	Yes	57	132	7,392
29		Yes	58	102	2,448
29		Yes	59	104	5,824

Overview of publication in TED. *peer_reviewed* indicates whether the publication was peer-reviewed at the time of inclusion in the database; *n_participants* shows the number of participants in a study; *n_trials* displays the overall number of trials in a study

**Fig. 4:**
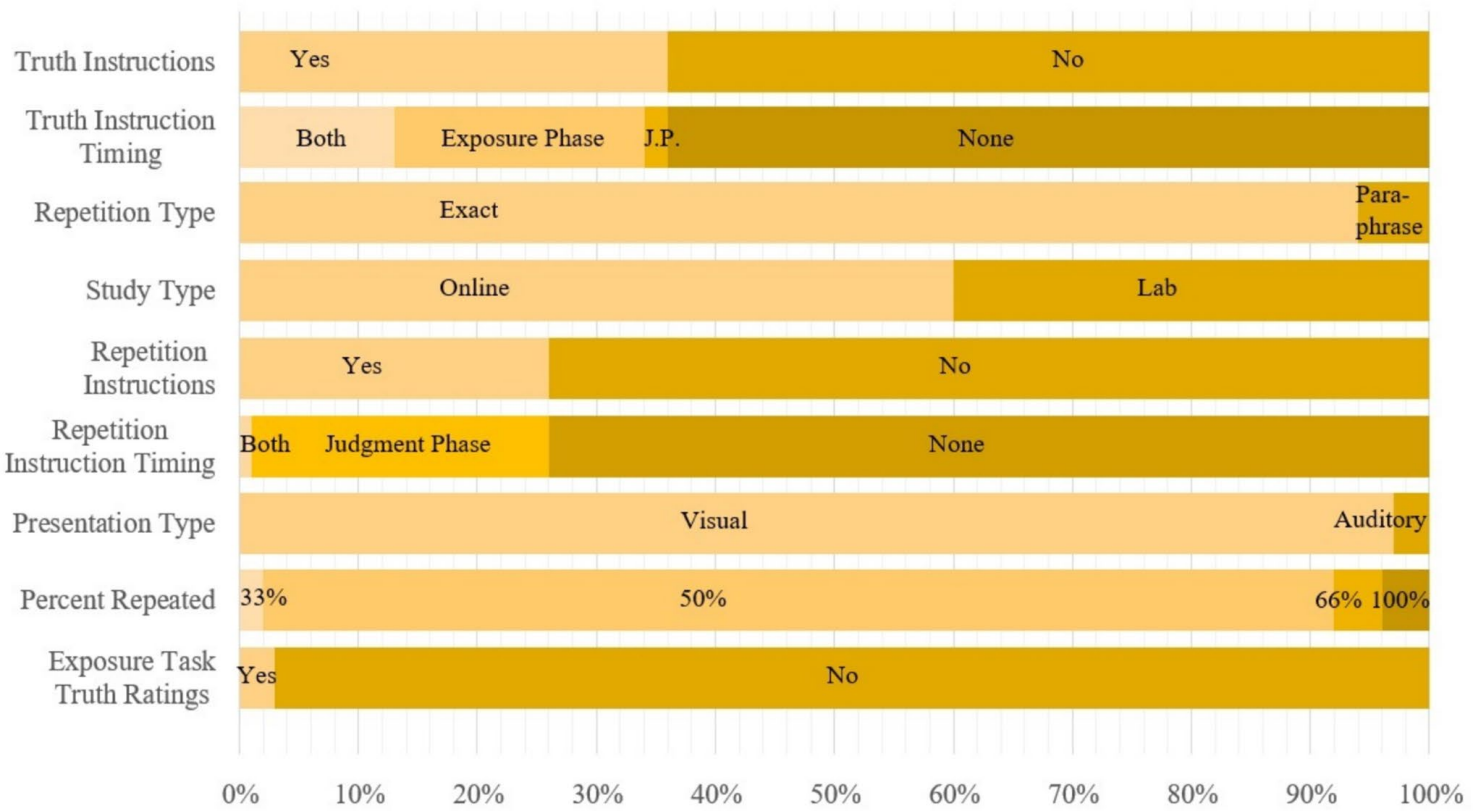
Overview of procedure-related variables in TED. *Note*. Overview of variables included in the *procedure_table* highlighting variability in additional ITE design features related to the experimental procedure across studies included in TED to date. The variables “Truth Instructions” and “Repetition Instructions” indicate whether participants were warned that some statements would be false or repeated, respectively. The variables “Truth Instruction Timing” and “Repetition Instruction Timing” specify the point in time (i.e., the experimental phase) when these warnings were given. The variables “Study Type” and “Repetition Type” indicate whether data were collected in the lab or online, and whether information was repeated exactly or paraphrased, respectively. “Presentation Type” indicates whether information was presented visually or auditorily, and “Percent Repeated” specifies the percentage of repeated statements (with 100% indicating that no new items were presented during the judgment phase, such that differences in truth ratings between the exposure and judgment phases were analyzed). “Exposure Task Truth Ratings” indicates whether participants were asked to rate the truth of statements during the exposure phase. Variable labels in the figure were slightly simplified for readability and may differ from the exact variable names in the database (e.g., “Truth Instructions” vs. truth_instructions)

On average, studies employed 62.71 ($SD=$ 38.66) statements per participant in the judgment session, and in 89.36% of procedure settings, exactly 50% of statements were repeated. Of 94 judgment phases, 73.40% were conducted on the same day as the exposure phase. The average delay between exposure and judgment phase if both were conducted on the same day was 4.04 min. The average delay between exposure and judgment phase, given the judgment phase was conducted at least 1 day after the exposure phase, was 7.40 days. An overview of additional variables pertaining to the procedure of the included studies can be found in Fig. 4.

Detailed information on the statements presented is available for 56 out of 59 studies. Data on the accuracy of a statement are available for 388,603 (54.38%) trials, the exact statement text is available for 326,877 (45.74%) trials, and response times are available for 140,567 (19.67%) trials.

### Bayesian multilevel modeling

To illustrate the benefits of our large collection of trial-level data, we fitted Bayesian multilevel models predicting truth judgments, with repetition as a fixed effect and random intercepts and slopes at the statement, subject, and experiment levels. We only included data from the test phase and excluded rows with missing values in the response or repetition status.

The experiment level is based on data from the *procedure_table*, as this table contains detailed information about each experimental setup (e.g., delay between exposure and test session, proportion of repeated items, presence of warnings) beyond what is available in the broader *study_table*. Each entry in the study_table corresponds to at least one entry in the *procedure_table*, but a single study may include several procedures that differ in these settings. For example, the same study may have multiple judgment sessions, modify the percentage of repeated stimuli, or warn some participants about the truth effect. These different procedures will then also receive different procedure identifiers but the same study identifier. Critically, this results in more “experiments” than studies included in TED. For example, while TED includes 59 studies, these studies consist of a total of 85 different procedural settings—“experiments”—analyzed in this paper.

Thus, the experiment identifier (*procedure_id*) uniquely captures both the study context and its specific experimental conditions. This modeling approach allows us to estimate the variance in the truth effect at three levels simultaneously: (1) variance due to diverse statements (statement level), (2) variance due to individual differences (subject level), and (3) variance due to common experimental manipulations and study settings (experiment level).

We analyzed the dichotomous and Likert-type response formats separately due to differences in their scale characteristics. Dichotomous responses (e.g., true/false) require logistic models, whereas Likert-scale responses (e.g., 1–5 ratings) allow for linear models. All responses were maximum-normalized to the range 0–1, with 1 representing the maximum possible response indicating a “true” judgment. The repetition status was effect coded with − 0.5 for new and + 0.5 for repeated statements.

We ran all models using four chains with 3,000 iterations per chain, 1,000 of which were discarded as warmup samples, leading to a total of 8,000 posterior samples. There were no divergent transitions, no $\widehat{R}>1.05$, and visual inspection confirmed that the chains mixed well. We used weakly informative priors for the intercept, fixed effect, and standard deviations (SD) for all models.

$$Intercept\sim Normal\left(0.5,0.5\right)$$

$$b\sim Normal\left(0,1\right)$$

$$\sigma \sim Gamma\left(1,4\right)$$

#### Dichotomous truth judgments

The analysis was based on 141,889 trials nested within 1,823 subjects, 1,357 statements, and 20 experiments.

Table 3 provides a summary of parameter estimates. As expected, the model indicated a credible fixed effect of repetition ($b= 0.63, 95\mathrm{\%}\hspace{0.25em}CrI=$ [0.49, 0.77], $B{F}_{10}>1\times {10}^{10}$, $OR=$ 1.87). Notably, the standard deviation of the random slope of repetition was highest at the subject level ($\sigma =$ 0.67, $95\mathrm{\%}\hspace{0.25em}CrI=$ [0.63, 0.71]), followed by the experiment level ($\sigma =$ 0.29, $95\mathrm{\%}\hspace{0.25em}CrI=$ [0.21, 0.42]) and the statement level ($\sigma =$ 0.16, $95\mathrm{\%}\hspace{0.25em}CrI=$ [0.11, 0.21]).

**Table 3:** Parameter estimates of the dichotomous model

Effect	Grouping	Parameter	Estimate	l_95_CrI	u_95_CrI
Fixed		Intercept	0.36	0.26	0.47
Fixed		Repeated	0.63	0.49	0.77
Random	Experiment	Intercept (SD)	0.17	0.10	0.27
Random	Experiment	Repeated (SD)	0.29	0.21	0.42
Random	Statement	Intercept (SD)	0.81	0.78	0.85
Random	Statement	Repeated (SD)	0.16	0.11	0.21
Random	Subject	Intercept (SD)	0.66	0.63	0.68
Random	Subject	Repeated (SD)	0.67	0.63	0.71

*N* = 141,889; no. experiments = 20; no. subjects = 1,823; no. statements = 1,357; l_95_CrI refers to the lower boundary of the 95% credible interval, u_95_CrI refers to the upper boundary

#### Scale truth judgments

The analysis was based on 572,775 trials nested within 8,309 subjects, 2,872 statements, and 66 experiments.

Table 4 provides a summary of parameter estimates. As expected, the model indicated a credible fixed effect of repetition ($b=$ 0.08, $95\mathrm{\%}\hspace{0.25em}CrI=$ [0.07, 0.10], $B{F}_{10}>1\times {10}^{10}$). Again, the standard deviation of the random slope of repetition was highest at the subject level ($\sigma =$ 0.10, $95\mathrm{\%}\hspace{0.25em}CrI=$ [0.10, 0.10]), followed by the experiment level ($\sigma =$ 0.07, $95\mathrm{\%}\hspace{0.25em}CrI=$ [0.05, 0.08]) and the statement level ($\sigma =$ 0.03, $95\mathrm{\%}\hspace{0.25em}CrI=$ [0.02, 0.03]).

**Table 4:** Parameter estimates of the scale model

Effect	Grouping	Parameter	Estimate	l_95_CrI	u_95_CrI
Fixed		Intercept	0.54	0.52	0.56
Fixed		Repeated	0.08	0.07	0.10
Random	Experiment	Intercept (SD)	0.07	0.06	0.09
Random	Experiment	Repeated (SD)	0.07	0.05	0.08
Random	Statement	Intercept (SD)	0.11	0.11	0.12
Random	Statement	Repeated (SD)	0.03	0.02	0.03
Random	Subject	Intercept (SD)	0.10	0.09	0.10
Random	Subject	Repeated (SD)	0.10	0.10	0.10

*N* = 572,775; no. experiments = 65; no. subjects = 8,309; no. statements = 2,872; l_95_CrI refers to the lower boundary of the 95% credible interval, u_95_CrI refers to the upper boundary

As outlined above in the Introduction, previous research findings suggest that the time interval between exposure and judgment phase may be a crucial factor in investigating the role of individual differences in the truth effect. To further explore the influence of temporal delay between the exposure and judgment phases on inter-individual variability in the repetition effect, we included an interaction between subject and temporal delay (same day vs. different day) in the random effect structure. The model then estimated two standard distributions for the random effect of repetition on the subject level. Based on this, we examined whether variability in the repetition effect at the subject level differs depending on the temporal delay.

**Fig. 5:**
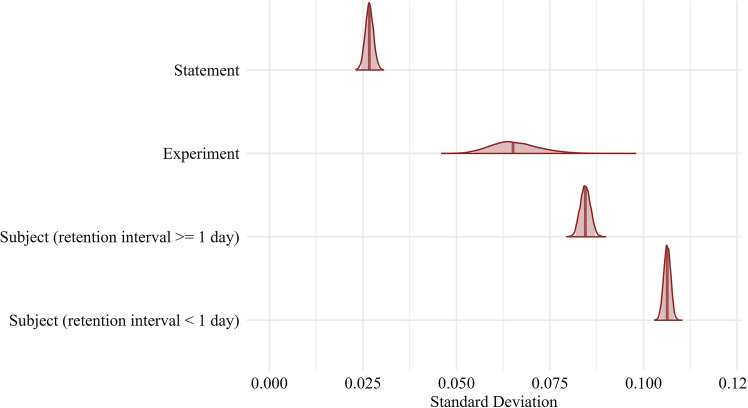
Variance in the truth effect at different levels. *Note.* Posterior distributions of the standard deviations of the random effects (i.e., variability) for the illusory truth effect at each hierarchical level. “Subject (Retention Interval < 1 day)” refers to judgment phases conducted on the same day as the exposure phase, and “Subject (Retention Interval ≥ 1 day) refers to judgment phases conducted at least one day after exposure

Table 5 provides a summary of parameter estimates. The standard deviation of the random slope for repetition at the subject level during the same-day judgment phase was ${\sigma }_{0}=$ 0.11 ($95\mathrm{\%}\hspace{0.25em}CrI=$ [0.10, 0.11]), whereas the standard deviation of the random slope during the later-day judgment phase was ${\sigma }_{1}=$ 0.08 ($95\mathrm{\%}\hspace{0.25em}CrI=$ [0.08, 0.09]). The difference in posterior distributions of the standard deviations in the random effect of repetition at the subject level deviated substantially from 0 ${(\sigma }_{0}-{\sigma }_{1}=$ 0.02 ($95\mathrm{\%}\hspace{0.25em}CrI=$ [0.02, 0.02], $B{F}_{10}>1\times {10}^{10}$) (Fig. 5).

**Table 5:** Parameter estimates of the time-based scale model

Effect	Grouping	Parameter	Estimate	l_95_CrI	u_95_CrI
fixed		Intercept	0.54	0.52	0.56
fixed		repeated	0.08	0.07	0.10
random	experiment	Intercept (SD)	0.07	0.06	0.09
random	experiment	repeated (SD)	0.07	0.05	0.08
random	statement	Intercept (SD)	0.11	0.11	0.12
random	statement	repeated (SD)	0.03	0.02	0.03
random	subject (same day)	Intercept (SD)	0.10	0.10	0.10
random	subject (same day)	repeated (SD)	0.11	0.10	0.11
random	subject (≥ 1 day)	Intercept (SD)	0.08	0.08	0.08
random	subject (≥ 1 day)	repeated (SD)	0.08	0.08	0.09

*N* = 572,775; no. experiments = 65; no. subjects = 8,309; no. statements = 2,872; l_95_CrI refers to the lower boundary of the 95% credible interval, u_95_CrI refers to the upper boundary

## Discussion

In this paper, we introduce the Truth Effect Database (TED), a large-scale, open-access repository of trial-level data on truth judgments. TED is designed to support cumulative, reproducible research on the illusory truth effect (ITE) by standardizing and centralizing data from a wide range of studies. It currently aggregates data from 59 studies in 29 publications, spanning 12,242 participants and 808,231 trials, accounting for a wide range of dispositional and contextual variables—including experimental instructions, task during initial exposure, number of repetitions, and length of the retention interval. With its growing size, TED provides a critical resource for investigating how repetition shapes belief formation—a process of increasing relevance in the digital age, where repeated exposure to false or misleading information is pervasive.

Our goal in developing TED was to address key challenges in open research data: the fragmentation of data across studies, the lack of standard formats for sharing experimental data, and the resulting lack of interoperability and reusability (Crüwell et al., 2023; Hardwicke et al., 2021). To meet these challenges, we provide a structured SQL-based schema that organizes data at multiple levels—including publications, experiments, participants, and individual trials. This structure allows for fine-grained analysis while maintaining consistency across diverse datasets.

We also provide tools to facilitate access and use of the database. In particular, the R package acdcquery (Lesche, 2025) allows users to extract data from TED with flexible filtering criteria, supporting both simple and complex queries. Critically, this package also provides functions to download the latest version of TED or check whether the installed version is up to date. Additionally, we have developed a web-based data entry interface that automatically checks for consistency and flags incorrect variable names or entries (https://slesche.github.io/truth-effect-database/). This helps maintain high standards for incoming data and ensures that TED remains a reliable resource for the community while simplifying the submission process and lowering the barrier to entry.

To illustrate the analytical potential of TED, we fitted multilevel models predicting truth judgments. By estimating random effects at the statement, subject, and experiment levels, we investigated where variance in the truth effect originates.

In both the Likert-scale and dichotomous truth judgment data, we found that the random slopes of repetition on the statement level showed very little variance. This suggests that specific properties of individual statements (e.g., their semantic content or plausibility) have limited influence on the magnitude of the truth effect. However, statement-level variance in random intercepts was relatively large. This is consistent with expectations: some statements are objectively easier to judge as true or false, while others are more ambiguous, leading to baseline differences in overall truth ratings.

We also observed substantial variance in the experiment-level random effect of repetition. This is unsurprising, as experiments varied considerably in design features such as the number of statements, delay between exposure and judgment phase, or whether participants were warned about repetition. Nevertheless, the experiment-level variance in the effect of repetition was still smaller than at the subject level.

In fact, the largest variance in the truth effect emerged at the subject level, indicating substantial individual differences in susceptibility to the repetition effect. This finding aligns with growing interest in understanding the psychological traits or cognitive mechanisms that moderate the truth effect at the individual level. However, efforts to study individual differences in the truth effect have so far produced mixed results (for an overview, see the Introduction).

In this exemplary use of TED, we furthermore explored whether the individual variance in the ITE is moderated by the delay between exposure and judgment phase—a key design feature in truth effect experiments. TED allowed us to perform an analysis using a large number of ITE datasets, estimating individual variability in the truth effect when the exposure and judgment occur on the same day, as opposed to a delay of at least 1 day, while additionally taking variance at the experiment and statement level into account. The results revealed that the variance in the repetition effect at the subject level was larger when truth judgments for repeated (vs. new) statements were made on the same day as the exposure phase, compared to when the judgment phases were postponed to a later date (i.e., at least 1 day later). Based on the posterior distributions, we compared these random effects between conditions (retention interval < 1 day vs. retention interval ≥ 1 day). Results demonstrated that this difference is different from 0, indicating credible differences in the subject-level variability in the illusory truth effect as a function of the delay between exposure and judgment phase. On a theoretical level, these differences as a function of retention interval length may reflect variations in memory strength. After a relatively short delay, memory traces are likely still robust, making it easier for individuals to identify the earlier presentation as the source of the familiarity experience. Arkes et al. (1989) found that the illusory truth effect increases when repeated statements are attributed to sources external to the experiment. We argue that after a relatively short delay, memory traces are likely still robust, making it easier for individuals to identify the earlier presentation as the source of the familiarity experience. Consequently, as long as the feeling of fluency can be traced back to repetition within the experimental context, certain dispositional factors may reduce the likelihood of relying on repetition-based fluency (e.g., a higher preference for deliberation, a lower need for cognitive closure). After longer intervals (e.g., 1 week), the source of familiarity likely becomes less distinct, reducing the interaction between repetition and dispositional factors. Once the familiarity can no longer be attributed to the experimental context, reliance on repetition-induced fluency may serve as a valid cue for truth evaluation under uncertainty (Reber & Unkelbach, 2010). This idea aligns well with prior research demonstrating that dispositional effects (e.g., need for cognitive closure) tend to emerge after relatively short retention intervals (e.g., 10 min) rather than longer intervals (e.g., 1 week; Stump et al., 2022).

Finally, although our findings align well with previous research, we emphasize that these preliminary results and theoretical assumptions should be interpreted with caution, as we did not control for potential confounding factors such as sample composition, procedural variation, or other study-level covariates. Overall, these initial analyses were primarily intended to demonstrate the usefulness of TED for addressing complex research questions that would be difficult to examine using data from a single study. The open and extensible nature of TED ensures that these questions can be revisited and refined as the database continues to grow. We see this as a first step toward more nuanced investigations of the truth effect grounded in large-scale, reproducible data.

### Limitations

Different limitations of the current version of TED should be acknowledged. First, TED is not a comprehensive repository of all truth effect studies. The dataset is based primarily on the systematic review by Henderson et al. (2022), and we have updated the database through our own literature searches. Consequently, any inferences drawn from analyses of TED should be interpreted in light of this selection bias: TED includes only those studies that have already made their data publicly available. To fully resolve issues of selection and interoperability, all original data would need to be made publicly accessible and well documented. Nevertheless, TED represents a step toward this goal and is uniquely positioned to facilitate data harmonization; the entry mask and harmonization efforts implemented in the database reduce barriers to interoperable data for future research.

Second, the submission process still requires contributors to send data directly to the maintainers via email. Although this procedure is relatively low-tech, it was deliberately designed to minimize points of failure and attack, avoid server hosting costs, and foster direct contact between contributors and maintainers, which can support follow-up and clarification. Similarly, TED currently does not provide an application programming interface (API) or server access. Instead, data are accessible via a downloadable file or directly through the accompanying R package, allowing researchers to interact with the database at their own pace and modify it as needed, while limiting project complexity and costs.

Third, the database is not static. Information may change over time as new studies are added, errors are corrected, or classifications are refined. In particular, the veracity of statements encoded in the database reflects their status at the time of data collection for the publication in which they were used. Because factual accuracy can change over time, researchers should exercise caution when using statement texts directly from TED and consider re-evaluating statement accuracy where necessary.

Expansion of the database is also constrained by available resources. In the absence of dedicated institutional support or direct funding, growth depends on voluntary submissions from researchers and on targeted survey efforts conducted by the maintenance team. We have aimed to make contributing to TED as straightforward as possible to lower barriers to participation. We also plan to periodically survey new research and reach out directly to potential contributors. All contributions help improve the “FAIR-ness” of illusory truth effect data and also increase the visibility of contributors’ work. We are also hopeful that broader initiatives promoting the development and use of large-scale databases, such as the recent call for submissions using large-scale data in *Psychometrika* (Psychometric Society, 2026), will further strengthen community support for TED.

Finally, because TED evolves over time, researchers should ensure that the version of the database they use matches the most current release. To facilitate this, the accompanying R package provides the check_ted() function, which allows users to compare a locally stored SQLite file with the latest version available online. If discrepancies are detected, users can update their local copy using update_ted() or download a specific release via download_ted(path, tag = "v1.1.0-example"), thereby ensuring transparency and reproducibility in analyses based on TED.

### Future research

TED offers a unique foundation for advancing research through open, cumulative, and reproducible science. As the database grows in scope and depth, we anticipate several promising directions for future research and methodological development that TED is particularly well positioned to support.

One immediate application lies in enabling living meta-analyses of truth effect data that can evolve as new studies are contributed. Importantly, the present data do not represent a random or comprehensive sample of all published ITE studies and therefore do not support formal meta-analysis. Nevertheless, TED can be used to estimate average effect sizes among studies with openly available data. Given these limitations, we chose not to report an aggregate effect size in the current paper. However, we provide a proof-of-concept meta-analysis on TED’s website (https://slesche.github.io/ted-site/explore.html#meta-analysis).

Closely related, TED can support customized power analyses based on empirical variance from real experimental data. Researchers designing new studies can use TED’s trial-level data to simulate expected effects based on previously collected data with similar study characteristics, improving the precision and efficiency of future study planning.

TED’s reproducibility potential lies in its ability to support both the replication and reanalysis of existing studies using openly available, trial-level data. Researchers can directly replicate earlier truth effect experiments by matching procedural details drawn from the database, or they can reanalyze existing datasets to test new hypotheses, apply alternative statistical models, or verify published results. Thus, TED facilitates both direct and conceptual replications and provides a foundation for transparent, data-driven reassessment of prior findings.

Beyond supporting replications, TED offers a foundation for formal modeling approaches to the truth effect. Its detailed trial-level data and metadata allow researchers to fit and validate cognitive process models, such as drift diffusion (see e.g., Ratcliff & McKoon, 2008) or multinomial processing tree models (see, e.g., Calio et al., 2020; Unkelbach & Stahl, 2009).

Importantly, these efforts are supported by TED’s intentional focus on long-term sustainability. The database is built with minimal reliance on proprietary infrastructure or paid software. All tools and data are hosted on open platforms, primarily GitHub, and contributions are made through transparent, version-controlled workflows. This lightweight architecture was chosen to promote longevity and ensure that TED remains accessible and modifiable regardless of funding cycles or institutional changes.

To ensure proper attribution and foster community investment, users of TED data are expected to cite all original publications corresponding to the datasets they use. In return, contributions to TED not only extend the utility of existing research but also amplify the visibility of individual studies, creating a mutually reinforcing incentive structure for both data sharing and reuse.

As TED continues to grow, we encourage researchers not only to contribute data but also to build on TED’s infrastructure itself. TED is an extension of the Attentional Control Data Collection (ACDC) project (Haaf et al., 2025), improving on data submission and ease of use. Both projects can serve as a jumping-off point for future database development. All aspects of our project, including the SQL schema, submission interface, R extraction package, and the interactive overview website, are open-source and fully forkable. Researchers are invited to reuse or adapt these components to develop similar infrastructures for other domains of psychological or behavioral science. In this way, TED can help establish a broader culture of open, scalable, and sustainable data practices in experimental research.

Taken together, these features position TED as a blueprint for cumulative science, one that supports rigorous replication, sophisticated cognitive modeling, empirical synthesis, and infrastructure reusability while lowering the barriers to collaboration and long-term research development.

## Conclusion

In this paper, we introduced the Truth Effect Database (TED), a structured, large-scale resource for cumulative research on truth judgments. TED includes not only standardized, trial-level data but also open tools for user interaction, including a submission interface and an R package for flexible data extraction. Additional details can be found on TED’s homepage (https://slesche.github.io/ted-site/).

We illustrated the utility of TED through Bayesian multilevel analyses, which highlighted substantial variance in the illusory truth effect at the subject level and provided new evidence that individual differences in susceptibility to the truth effect systematically vary as a function of the retention interval length. These findings point to the need for further theoretical and empirical work on individual differences in susceptibility to information repetition.

Beyond this first demonstration, TED provides the foundation for a wide range of future research. These include (living) meta-analyses, simulation-based power analyses, rigorous replication and reanalysis of existing studies, and the development of formal cognitive models. As an open and extensible infrastructure, TED also serves as a blueprint for sustainable, community-driven database development in psychological science.

## Data Availability

All data and code needed to replicate this work are publicly available at: 10.5281/zenodo.17592982. The overview website for the database can be found at https://slesche.github.io/ted-site, the submission tool can be found at https://github.com/SLesche/truth-effect-database, and the extraction package can be found at https://github.com/SLesche/acdc-query.

## Footnotes

1 We also used the R packages *acdcquery* (Version 1.0.1; Lesche, 2025), *brms* (Version 2.22.0; Bürkner, 2017, 2018, 2021), *knitr* (Version 1.50; Xie, 2015), *papaja* (Version 0.1.3; Aust & Barth, 2022), *rstan* (Version 2.32.6; Stan Development Team, 2024), and *tidyverse* (Version 2.0.0; Wickham et al., 2019).

## Code availability

All data and code needed to replicate this work are publicly available at 10.5281/zenodo.17592982.

## Declarations

### Open practices statement

None of the reported analyses were preregistered. All data and code needed to replicate this work is publicly available at 10.5281/zenodo.17592982. The overview website for the database can be found at https://slesche.github.io/ted-site, the submission tool can be found at https://github.com/SLesche/truth-effect-database, and the extraction package can be found at https://github.com/SLesche/acdc-query.

## References

[CR1] Arkes, H. R., Boehm, L. E., & Xu, G. (1991). Determinants of judged validity. *Journal of Experimental Social Psychology*, *27*(6), 576–605.

[CR2] Arkes, H. R., Hackett, C., & Boehm, L. (1989). The generality of the relation between familiarity and judged validity. *Journal of Behavioral Decision Making,**2*(2), 81–94. 10.1002/bdm.3960020203

[CR3] Aust, F., & Barth, M. (2022). *papaja: Prepare reproducible APA journal articles with R Markdown*. https://github.com/crsh/papaja

[CR4] Bauer, P. J. (2022). *Psychological science stepping up a level* (2. *SAGE Publications Sage CA: Los Angeles, CA,**33*, 179–183. 10.1177/09567976221078527 10.1177/09567976221078527 35102774

[CR5] Béna, J., Carreras, O., Terrier, P. (2019*). Attention division and the truth effect: A case of moderation by source credibility manipulation.* Unpublished.

[CR6] Béna, J., Carreras, O., Terrier, P. (2020*). Does delay between exposure and truth judgement decrease the truth effect through a recollection impairment? A Remember/Know study".* Unpublished data

[CR7] Béna, J., Corneille, O., Mierop, A., & Unkelbach, C. (2022). Robustness tests replicate Corneille et al.’s (2020) fake news by repetition effect. *International Review of Social Psychology*, *35*(1).

[CR8] Boehm, L. E. (1994). The validity effect: A search for mediating variables. *Personality and Social Psychology Bulletin,**20*(3), 285–293. 10.1177/0146167294203006

[CR9] Brashier, N. M., Eliseev, E. D., & Marsh, E. J. (2020). An initial accuracy focus prevents illusory truth. *Cognition,**194*, Article 104054. 10.1016/j.cognition.2019.104054 31473395 10.1016/j.cognition.2019.104054

[CR10] Bürkner, P.-C. (2017). brms: An R package for Bayesian multilevel models using Stan. *Journal of Statistical Software,**80*(1), 1–28. 10.18637/jss.v080.i01

[CR11] Bürkner, P.-C. (2018). Advanced Bayesian multilevel modeling with the R package brms. *The R Journal,**10*(1), 395–411. 10.32614/RJ-2018-017

[CR12] Bürkner, P.-C. (2021). Bayesian item response modeling in R with brms and Stan. *Journal of Statistical Software,**100*(5), 1–54. 10.18637/jss.v100.i05

[CR13] Calio, F., Nadarevic, L., & Musch, J. (2020). How explicit warnings reduce the truth effect: A multinomial modeling approach. *Acta Psychologica,**211*, Article 103185. 10.1016/j.actpsy.2020.103185 33130489 10.1016/j.actpsy.2020.103185

[CR14] Crüwell, S., Apthorp, D., Baker, B. J., Colling, L., Elson, M., Geiger, S. J., Lobentanzer, S., Monéger, J., Patterson, A., Schwarzkopf, D. S., et al. (2023). What’s in a badge? A computational reproducibility investigation of the open data badge policy in one issue of Psychological Science. *Psychological Science,**34*(4), 512–522. 10.1177/09567976221140828 36730433 10.1177/09567976221140828

[CR15] Dechêne, A., Stahl, C., Hansen, J., & Wänke, M. (2009). Mix me a list: Context moderates the truth effect and the mere-exposure effect. *Journal of Experimental Social Psychology,**45*(5), 1117–1122. 10.1016/j.jesp.2009.06.019

[CR16] Dechêne, A., Stahl, C., Hansen, J., & Wänke, M. (2010). The truth about the truth: A meta-analytic review of the truth effect. *Personality and Social Psychology Review,**14*(2), 238–257. 10.1177/1088868309352251 20023210 10.1177/1088868309352251

[CR17] De Keersmaecker, J., Dunning, D., Pennycook, G., Rand, D. G., Sanchez, C., Unkelbach, C., & Roets, A. (2020). Investigating the robustness of the illusory truth effect across individual differences in cognitive ability, need for cognitive closure, and cognitive style. *Personality and Social Psychology Bulletin,**46*(2), 204–215. 10.1177/0146167219853844 31179863 10.1177/0146167219853844

[CR18] De keersmaecker, J., Unkelbach, C., & Roets, A. (2024). Truth-by-repetition across languages.. *Journal of Applied Research in Memory and Cognition.* Advance online publication. 10.1037/ mac0000175

[CR19] De Keersmaecker, J., Wiernik, B. M., Roets, A., & Unkelbach, C. (2025). Truth by repetition reliably differs between people over time. 10.31234/osf.io/aeukr_v1

[CR20] Deutsche Forschungsgemeinschaft. (2025, 3. April). *Förderprogramm „Informationsinfrastrukturen für Forschungsdaten“*. https://www.dfg.de/de/foerderung/foerdermoeglichkeiten/programme/infrastruktur/lis/lis-foerderangebote/forschungsdaten

[CR21] DiFonzo, N., Beckstead, J. W., Stupak, N., & Walders, K. (2016). Validity judgments of rumors heard multiple times: The shape of the truth effect. *Social Influence,**11*(1), 22–39. 10.1080/15534510.2015.1137224

[CR22] Ecker, U., Lewandowsky, S., & Chadwick, M. (2020). *Can corrections spread misinformation to new audiences? Testing for the elusive familiarity backfire effect.*10.31234/osf.io/qrm69 10.1186/s41235-020-00241-6 PMC7447737 32844338

[CR23] Fazio, L. K., Rand, D. G., & Pennycook, G. (2019). Repetition increases perceived truth equally for plausible and implausible statements. *Psychonomic Bulletin & Review,**26*(5), 1705–1710. 10.3758/s13423-019-01651-4 31420808 10.3758/s13423-019-01651-4

[CR24] Foster, E. D., & Deardorff, A. (2017). Open Science Framework (OSF). *Journal of the Medical Library Association*, *105*(2). 10.5195/jmla.2017.88

[CR25] Gorgolewski, K. J., Auer, T., Calhoun, V. D., Craddock, R. C., Das, S., Duff, E. P., Flandin, G., Ghosh, S. S., Glatard, T., Halchenko, Y. O., et al. (2016). The brain imaging data structure, a format for organizing and describing outputs of neuroimaging experiments. *Scientific Data,**3*(1), 1–9. 10.1038/sdata.2016.44 10.1038/sdata.2016.44 PMC4978148 27326542

[CR26] Haaf, J. M., Hoffstadt, M., & Lesche, S. (2025). Attentional control data collection: A resource for efficient data reuse. *Behavior Research Methods,**57*(8), Article 208. 10.3758/s13428-025-02717-z 40555897 10.3758/s13428-025-02717-z PMC12187800

[CR27] Hardwicke, T. E., Bohn, M., MacDonald, K., Hembacher, E., Nuijten, M. B., Peloquin, B. N., DeMayo, B. E., Long, B., Yoon, E. J., & Frank, M. C. (2021). Analytic reproducibility in articles receiving open data badges at the journal Psychological Science: An observational study. *Royal Society Open Science,**8*(1), Article 201494. 10.1098/rsos.201494 33614084 10.1098/rsos.201494 PMC7890505

[CR28] Hasher, L., Goldstein, D., & Toppino, T. (1977). Frequency and the conference of referential validity. *Journal of Verbal Learning and Verbal Behavior,**16*(1), 107–112. 10.1016/S0022-5371(77)80012-1

[CR29] Hatzidaki, A., Santesteban, M., & Navarrete, E. (2025). Illusory truth effect across languages and scripts. *Psychonomic Bulletin & Review,**32*(3), 1231–1239. 10.3758/s13423-024-02596-z 39466589 10.3758/s13423-024-02596-z

[CR30] Henderson, E. L., Simons, D. J., & Barr, D. J. (2021). The trajectory of truth: A longitudinal study of the illusory truth effect. *Journal of Cognition,**4*(1), Article 29. 10.5334/joc.161 34164597 10.5334/joc.161 PMC8194981

[CR31] Henderson, E. L., Westwood, S. J., & Simons, D. J. (2022). A reproducible systematic map of research on the illusory truth effect. *Psychonomic Bulletin & Review,**29*(3), 1065–1088. 10.3758/s13423-021-01995-w 34708397 10.3758/s13423-021-01995-w PMC9166874

[CR32] Hipp, R. D. (2020). *SQLite* (Version 3.31.1).

[CR33] Ioannidis, J. P. (2005). Why most published research findings are false. *PLoS Medicine,**2*(8), Article e124. 10.1371/journal.pmed.0020124 16060722 10.1371/journal.pmed.0020124 PMC1182327

[CR34] Jalbert, M., Newman, E., & Schwarz, N. (2020). Only half of what i’ll tell you is true: Expecting to encounter falsehoods reduces illusory truth. *Journal of Applied Research in Memory and Cognition,**9*(4), 602–613. 10.1016/j.jarmac.2020.08.010

[CR35] Kim, C. (2002). *The role of individual differences in general skepticism in the illusory truth effect* [Doctoral dissertation, University of Cincinnati]. ProQuest. https://search.proquest.com/docview/304789925

[CR36] Lacassagne, D., Béna, J., & Corneille, O. (2022). Is earth a perfect square? Repetition increases the perceived truth of highly implausible statements. *Cognition,**223*, Article 105052. 10.1016/j.cognition.2022.105052 35144111 10.1016/j.cognition.2022.105052

[CR37] Lesche, S. (2025). *Acdcquery: Query the attentional control data collection*. https://github.com/SLesche/acdc-query 10.3758/s13428-025-02717-z PMC12187800 40555897

[CR38] Lonsdorf, T. B., & Ehlers, M. R. (2025, April 5). curated data collection (the FEAR BASE). Retrieved from osf.io/rkp3a

[CR39] Lorenzoni, A., Faccio, R., & Navarrete, E. (2024). Does foreign-accented speech affect credibility? Evidence from the illusory-truth paradigm. *Journal of Cognition,**7*(1), Article 26. 10.5334/joc.353 38405636 10.5334/joc.353 PMC10885845

[CR40] Munafò, M. R., Nosek, B. A., Bishop, D. V., Button, K. S., Chambers, C. D., Percie du Sert, N., Simonsohn, U., Wagenmakers, E.-J., Ware, J. J., & Ioannidis, J. P. (2017). A manifesto for reproducible science. *Nature Human Behaviour,**1*(1), Article 0021. 10.1038/s41562-016-0021 33954258 10.1038/s41562-016-0021 PMC7610724

[CR41] Murray, S., Stanley, M., McPhetres, J., Pennycook, G., & Seli, P. (2020). "I’ve said it before and I will say it again": Repeating statements made by Donald Trump increases perceived truthfulness for individuals across the political spectrum. 10.31234/osf.io/9evzc

[CR42] Nadarevic, L. (2007*). A failed replication of the truth effect.* Unpublished raw data. 10.17605/OSF.IO/FW2QE

[CR43] Nadarevic, L. (2010). *Die Wahrheitsillusion [The illusory truth effect]*. Verlag Dr. Köster.

[CR44] Nadarevic, L. & Rinnewitz, L. (2011). *Judgment mode instructions do not moderate the truth effect.* Unpublished raw data. 10.17605/OSF.IO/3UAJ7

[CR45] Nadarevic L., Meckler D., & Schmidt, A. (2012). *An investigation of the truth effect and different personality traits.* Unpublished raw data. 10.17605/OSF.IO/6WV4Z

[CR46] Nadarevic, L., & Erdfelder, E. (2014). Initial judgment task and delay of the final validity-rating task moderate the truth effect. *Consciousness and Cognition,**23*, 74–84. 10.1016/j.concog.2013.12.002 24370608 10.1016/j.concog.2013.12.002

[CR47] Nadarevic, L., & Aßfalg, A. (2017). Unveiling the truth: Warnings reduce the repetition-based truth effect. *Psychological Research = Psychologische Forschung,**81*, 814–826. 10.1007/s00426-016-0777-y 27318939 10.1007/s00426-016-0777-y

[CR48] Nadarevic, L., Plier, S., Thielmann, I., & Darancó, S. (2018). Foreign language reduces the longevity of the repetition-based truth effect. *Acta Psychologica,**191*, 149–159. 10.1016/j.actpsy.2018.08.019 30273765 10.1016/j.actpsy.2018.08.019

[CR49] Nadarevic, L., Reber, R., Helmecke, A. J., & Köse, D. (2020). Perceived truth of statements and simulated social media postings: An experimental investigation of source credibility, repeated exposure, and presentation format. *Cognitive Research: Principles and Implications,**5*(1), 1–16. 10.1186/s41235-020-00251-4 33175284 10.1186/s41235-020-00251-4 PMC7656226

[CR50] Nadarevic, L., Schnuerch, M., & Stegemann, M. J. (2021). Judging fast and slow: The truth effect does not increase under time-pressure conditions. *Judgment and Decision Making,**16*(5), 1234–1266.

[CR51] Nadarevic, L., & Erdfelder, E. (2025). On the relationship between recognition judgments and truth judgments: Memory states moderate the recognition-based truth effect. *Journal of Experimental Psychology: Learning, Memory, and Cognition.*10.31234/osf.io/2vzpx_v2 10.1037/xlm0001460 39964518

[CR52] Newman, E. J., Jalbert, M. C., Schwarz, N., & Ly, D. P. (2020). Truthiness, the illusory truth effect, and the role of need for cognition. *Consciousness and Cognition,**78*, Article 102866. 10.1016/j.concog.2019.102866 31935624 10.1016/j.concog.2019.102866

[CR53] Nosek, B. A., Alter, G., Banks, G. C., Borsboom, D., Bowman, S. D., Breckler, S. J., Buck, S., Chambers, C. D., Chin, G., Christensen, G., et al. (2015). Promoting an open research culture. *Science,**348*(6242), 1422–1425. 10.1126/science.aab2374 26113702 10.1126/science.aab2374 PMC4550299

[CR54] Pennycook, G., Cannon, T. D., & Rand, D. G. (2018). Prior exposure increases perceived accuracy of fake news. *Journal of Experimental Psychology: General,**147*(12), 1865. 10.1037/xge0000465 30247057 10.1037/xge0000465 PMC6279465

[CR55] Poldrack, R. A., Markiewicz, C. J., Appelhoff, S., Ashar, Y. K., Auer, T., Baillet, S., Bansal, S., Beltrachini, L., Benar, C. G., Bertazzoli, G., et al. (2024). The past, present, and future of the brain imaging data structure (BIDS). *Imaging Neuroscience,**2*, 1–19. 10.1162/imag_a_00103 39308505 10.1162/imag_a_00103 PMC11415029

[CR56] Psychometric Society. (2026, January 15). *Call for Papers: Psychometrika Special Issue — Data Intensive Methods in Psychometrics*. Retrieved [19.02.2026], from https://www.psychometricsociety.org/post/call-special-issue-intensive-methods

[CR57] R Core Team. (2022). *R: A language and environment for statistical computing*. R Foundation for Statistical Computing. https://www.R-project.org/

[CR58] Ratcliff, R., & McKoon, G. (2008). The diffusion decision model: Theory and data for two-choice decision tasks. *Neural Computation,**20*(4), 873–922. 10.1162/neco.2008.12-06-420 18085991 10.1162/neco.2008.12-06-420 PMC2474742

[CR59] Reber, R., & Unkelbach, C. (2010). The epistemic status of processing fluency as source for judgments of truth. *Review of Philosophy and Psychology,**1*, 563–581. 10.1007/s13164-010-0039-7 22558063 10.1007/s13164-010-0039-7 PMC3339024

[CR60] Schnuerch, M., Nadarevic, L., & Rouder, J. N. (2021). The truth revisited: Bayesian analysis of individual differences in the truth effect. *Psychonomic Bulletin & Review,**28*(3), 750–765. 10.3758/s13423-020-01814-8 33104997 10.3758/s13423-020-01814-8 PMC8219594

[CR61] Stan Development Team. (2024). *RStan: The R interface to Stan*. https://mc-stan.org/

[CR62] Stump, A., Rummel, J., & Voss, A. (2022). Is it all about the feeling? Affective and (meta-) cognitive mechanisms underlying the truth effect. *Psychological Research = Psychologische Forschung,**86*, 12–36. 10.1007/s00426-020-01459-1 33484352 10.1007/s00426-020-01459-1 PMC8821071

[CR63] Stump, A., Voss, A., & Rummel, J. (2024). The illusory certainty: Information repetition and impressions of truth enhance subjective confidence in validity judgments independently of the factual truth. *Psychological Research = Psychologische Forschung,**88*(4), 1288–1297. 10.1007/s00426-024-01956-7 38526581 10.1007/s00426-024-01956-7 PMC11143013

[CR64] Sundar, A., Kardes, F. R., & Wright, S. A. (2015). The influence of repetitive health messages and sensitivity to fluency on the truth effect in advertising. *Journal of Advertising,**44*(4), 375–387. 10.1080/00913367.2015.1045154

[CR65] Unkelbach, C., & Stahl, C. (2009). A multinomial modeling approach to dissociate different components of the truth effect. *Consciousness and Cognition,**18*(1), 22–38. 10.1016/j.concog.2008.09.006 18980847 10.1016/j.concog.2008.09.006

[CR66] Unkelbach, C., & Greifeneder, R. (2018). Experiential fluency and declarative advice jointly inform judgments of truth. *Journal of Experimental Social Psychology,**79*, 78–86. 10.1016/j.jesp.2018.06.010

[CR67] Vogel, T., Silva, R. R., Thomas, A., & Wänke, M. (2020). Truth is in the mind, but beauty is in the eye: Fluency effects are moderated by a match between fluency source and judgment dimension. *Journal of Experimental Psychology: General*. 10.1037/xge0000731 31944810 10.1037/xge0000731

[CR68] Wickham, H., Averick, M., Bryan, J., Chang, W., McGowan, L. D., François, R., Grolemund, G., Hayes, A., Henry, L., Hester, J., Kuhn, M., Pedersen, T. L., Miller, E., Bache, S. M., Müller, K., Ooms, J., Robinson, D., Seidel, D. P., Spinu, V., & Yutani, H. (2019). Welcome to the tidyverse. *Journal of Open Source Software,**4*(43), Article 1686. 10.21105/joss.01686

[CR69] Wilkinson, M. D., Dumontier, M., Aalbersberg, I. J., Appleton, G., Axton, M., Baak, A., Blomberg, N., Boiten, J.-W., da Silva Santos, L. B., Bourne, P. E., et al. (2016). The FAIR guiding principles for scientific data management and stewardship. *Scientific Data,**3*(1), 1–9. 10.1038/sdata.2016.18 10.1038/sdata.2016.18 PMC4792175 26978244

[CR70] Xie, Y. (2015). *Dynamic documents with R and knitr* (2nd ed.). Chapman; Hall/CRC. https://yihui.org/knitr/10.1201/b15166

